# Z-Spectrum Analysis Provides Proton Environment Data (ZAPPED): A New Two-Pool Technique for Human Gray and White Matter

**DOI:** 10.1371/journal.pone.0119915

**Published:** 2015-03-13

**Authors:** Mitsue Miyazaki, Cheng Ouyang, Xiangzhi Zhou, James B. Murdoch, Yasutaka Fushimi, Tomohisa Okada, Koji Fujimoto, Aki Kido, Yoshiki Arakawa, Susumu Miyamoto, Kaori Togashi

**Affiliations:** 1 MR Research, Toshiba Medical Research Institute, Vernon Hills, Illinois, United States of America; 2 Department of Diagnostic Imaging and Nuclear Medicine, Graduate School of Medicine, Kyoto University, Kyoto, Japan; 3 Department of Neurosurgery, Graduate School of Medicine, Kyoto University, Kyoto, Japan; Osaka University Graduate School of Medicine, JAPAN

## Abstract

A new technique – Z-spectrum Analysis Provides Proton Environment Data (ZAPPED) – was used to map cross-relaxing free and restricted protons in nine healthy subjects plus two brain tumor patients at 3T. First, MT data were acquired over a wide symmetric range of frequency offsets, and then a trio of quantitative biomarkers, i.e., the apparent spin-spin relaxation times (T_2,f_, T_2,r_) in both free and restricted proton pools as well as the restricted pool fraction F_r_, were mapped by fitting the measured Z-spectra to a simple two-Lorentzian compartment model on a voxel-by-voxel basis. The mean restricted exchangeable proton fraction, F_r_, was found to be 0.17 in gray matter (GM) and 0.28 in white matter (WM) in healthy subjects. Corresponding mean values for apparent spin-spin relaxation times were 785 µs (T_2,f_) and 17.7 µs (T_2,r_) in GM, 672 µs (T_2,f_) and 23.4 µs (T_2,r_) in WM. The percentages of F_f_ and F_r_ in GM are similar for all ages, whereas F_r_ shows a tendency to decrease with age in WM among healthy subjects. The patient ZAPPED images show higher contrast between tumor and normal tissues than traditional T_2_-weighted and T_1_-weighted images. The ZAPPED method provides a simple phenomenological approach to estimating fractions and apparent T_2_ values of free and restricted MT-active protons, and it may offer clinical useful information.

## Introduction

Magnetization transfer contrast (MTC), a mechanism that is used to indirectly detect macromolecular properties in magnetic resonance imaging (MRI) by observing the exchange of magnetization between "free" and "restricted" water protons in macromolecules, was originally introduced over 20 years ago [1]. MTC effects vary in human tissues and organs due to different macromolecular compositions therein; as a result, MRI contrast can be generated among these tissues by taking advantage of the different proton exchange levels. The well-known MTC effect has proven beneficial in evaluating the morphology of brain, kidney, and myocardium, as well as improving the contrast between blood and brain parenchyma in intracranial MR angiography [1–5].

The MTC effect has also been used to study specific exchangeable protons resonating at different frequencies by selectively saturating them and observing the resulting change in the free water signal, a technique known as chemical exchange saturation transfer (CEST) [6,7]. In CEST, protons unobservable in MR spectroscopy due to short lifetime or fast exchange are irradiated by off-resonance RF pulses and observed indirectly in the Z-spectra (i.e., signal observed on resonance as a function of the selective irradiation frequency). However, conventional CEST is used to observe the relative density of H-groups of interest at specific chemical shifts, and the usual offset frequency range (±1000 Hz) does not cover the broad spectrum of ultra-short T_2_ components far greater than ±1000 Hz in width.

Wide range Z-spectra have been investigated using a super-Lorentzian lineshape with acquisition of half the frequency range [8–10]. In this work, we analyzed Z-spectra acquired over a broad and symmetric range of frequency offsets by fitting them to a two Lorentzian compartment model, without the need to consider the detailed kinetics of the exchange process. The technique we propose—Z-spectrum Analysis Provides Proton Environment Data (ZAPPED)—allows us to map and extract important properties and information in both the free and restricted exchangeable proton pools, including the proton fractions (F_r_ and F_f_ = 1—F_r_) and *apparent* spin-spin relaxation times (T_2,f_, T_2,r_) derived from fitted Lorentzian linewidths. (Here “exchangeable” is short for “subject to magnetization transfer”—whether by dipolar coupling or actual chemical exchange.) With this simple imaging and analysis method, both ultra-short and long spin-spin relaxations can be quantified, even though the ultra-short restricted component is not directly observable in MR spectroscopy. We then expanded this method to investigate how these two exchangeable proton environments affect the MRI contrast of gray matter (GM) and white matter (WM) in segmented human brain, and further investigated the potential of this novel MT technique in clinical applications, such as brain tumor imaging.

## Materials and Methods

### Ethics Statement

The study was approved by both ethical review boards of Ethics Committee of Kyoto University Graduate School and Faculty of Medicine and of E&I Review Services for Toshiba Medical Research Institute USA, Inc. A written informed consent was obtained from all participants according to the guidance of the review boards of Kyoto University Graduate School and Faculty of Medicine and Toshiba Medical Research Institute USA, Inc., prior to their inclusion in the present study.

### Human Subjects

Z-spectra were acquired from nine healthy volunteers (7 males and 2 females; age range, 33–71 years old, and mean age, 47 years old) plus two patients (84-year-old male with malignant lymphoma and 26-year-old male with low-grade glioma). MR experiments were performed on 3 T systems (Vantage Titan 3T, Toshiba Medical Systems, Japan) using a standard body coil for transmission and a thirteen-channel head-array receive coil, following a protocol approved by the Institutional Review Boards of the institution. Padding was used to stabilize the subject’s head and to reduce movement during the scan.

### Imaging Experiments

Unlike typical CEST Z-spectrum acquisitions in which the selective saturation frequencies usually range from-5 to 5 ppm, we applied MT saturation pulses in 53 steps over an expanded range of off-resonance frequencies from-30 KHz to 30 KHz, with increments varying from 3000 Hz at the outer limits to 50 Hz near resonance. To maximize the MT effect while staying within the normal specific absorption rate (SAR) limit, ten narrowband 40-ms saturation pulses (sinc pulses with three pairs of sidelobes), each with a flip angle of 500°, were strung together, resulting in a total saturation duration of 400 ms and an overall saturation bandwidth (full width at half-maximum) of ~400 Hz. The MTC pulses were inserted as preparation pulses into a single-shot fast-spin-echo 2D sequence with TR/TE = 8553/60 ms. The excitation and refocus pulse angles were both 90^o^. The in-plane resolution was 1.0 mm x 1.1 mm, and the slice thickness was 5 mm. A single imaging slice was acquired for each human subject, and the total Z-spectrum acquisition time for each slice was around 7:30.

For all nine healthy human subjects, the imaging slice was placed axially, just below the body of the corpus callosum. For one of the nine subjects, two more acquisitions were performed at two different imaging positions: an axial slice passing through the third ventricle (at the basal ganglia level) and a tilted slice through the cerebellum. The purpose of these two scans was to confirm that the ZAPPED method proposed in our study was robust with respect to imaging location. For the two patients, the imaging slice was prescribed passing through the brain tumor with guidance from an experienced neuroradiologist.

### Curve-fitting Model and Parameter Estimation

As suggested [8], the MT effect of biological systems can be modeled using a Lorentzian lineshape. Since we hypothesize that there are at least two exchangeable proton pools, our two-component model for the Z-spectrum can be described by the following equation:

$$y\;=\;F_{f}\frac{{LW}_{f}^{2}}{{LW}_{f}^{2}+4x^{2}}+F_{r}\frac{{LW}_{r}^{2}}{{LW}_{r}^{2}+4x^{2}}$$ (1)

where *y* stands for the normalized measured Z-spectrum and *x* is the offset frequency of the MTC saturation pulses, ranging from-30 KHz to 30 KHz. F_f_ and F_r_ represent amplitudes or fractions of the free and restricted exchangeable components. Note that F_f_ + F_r_ = 1. LW_f_ and LW_r_ in units of Hz are full-widths at half maximum (FWHM) of these two components. In turn, the LW_f_ and LW_r_ values are inversely proportional to apparent spin-spin relaxation times: LW_f_ = 1 / (πT_2,f_) and LW_r_ = 1 / (πT_2,r_). Note that in order to simplify our ZAPPED model, the kinetics of the exchange process between the two pools is not explicitly included in Equation (1). As a result, the apparent relaxation times (T_2,f_ and T_2,r_) are weighted by other factors in addition to the transverse relaxation time itself, such as the exchange rate and longitudinal relaxation times. Moreover, the value of T_2,f_ is limited by the frequency resolution of the Z-spectrum. As a consequence, it is expected that the estimated apparent T_2,f_ and T_2,r_ values could be different from traditional transverse relaxation times (T_2_) of non-exchangeable water protons.

To estimate F_f_, F_r,_ LW_f_, and LW_r_ the acquired MT images were curve-fitted to the signal model in Equation (1), using the *lsqnonlin* function in MATLAB (MathWorks, Inc., Natick, MA), which solves nonlinear least-squares problems [11,12]. Gaussian and Lorentzian lineshapes were tried for both the free and restricted components, but the Lorentzian-Lorentzian combination yielded the best least-squares fit. We processed the data in two different ways. In the first approach, three tissue components, i.e., GM, WM and cerebrospinal fluid (CSF), were segmented in SPM8 (Statistical Parametric Mapping, www.fil.ion.ucl.ac.uk/spm/) using the Z-spectrum image at 30KHz offset frequency (which has minimal MT perturbation) as the input. The segmentation in SPM is a model-based algorithm using Bayesian rules to assign the probability for each voxel belonging to each tissue component [13]. The mean signal for each tissue component was calculated and then served as input in Equation (1). In the second approach, to obtain the ZAPPED maps, Z-spectra were curve-fitted using the two Lorentzian lineshapes in a voxel-by-voxel fashion.

## Results

**Fig 1:**
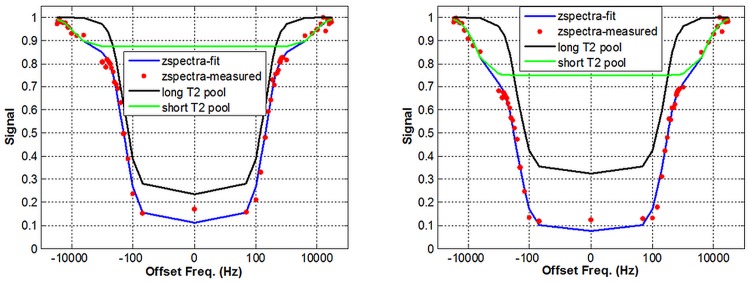
Measured Z-spectra (red) of GM (left) and WM (right), and fitted curves (blue) on a logarithmic scale. The model assumes that there are two exchangeable proton components: restricted (green) and free (black).

Gray matter, white matter, and CSF were successfully segmented for all nine subjects. The measured mean signals of GM and WM for each subject were fitted with Lorentzian lineshapes, and the fitted Z-spectra matched the experimental ones very well. Fig. 1 shows an example of the curve-fitting of the free and restricted exchangeable components in GM (left) and WM (right) on a logarithmic frequency scale from one volunteer. The red curve represents the acquired Z-spectrum, and the blue one is the fitted Z-spectrum using the dual-Lorentzian model. The fitted free and restricted exchangeable components are shown separately in black and green. The normalized-root-mean-square difference (NRMSD) was 0.02 for the GM fit and 0.03 for the WM fit.

**Fig 2:**
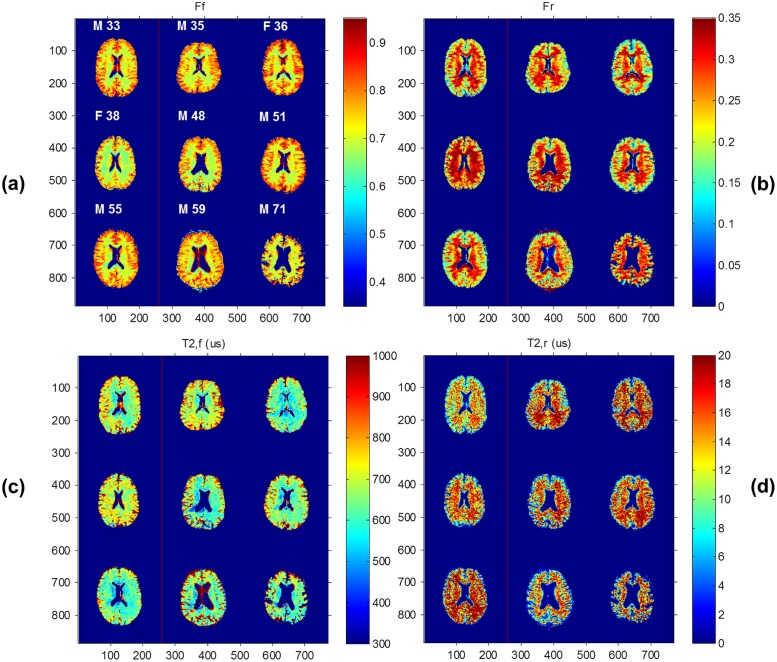
Color maps of nine healthy volunteers (a) Fraction map of the free exchangeable component, F_f_, (b) Fraction of the restricted exchangeable component, F_r_, (c) T_2,f_ map, and (d) T_2,r_ map. Note that the F_r_ maps are just reflections of the F_f_ maps since F_r_ + F_f_ = 1.

**Fig 3:**
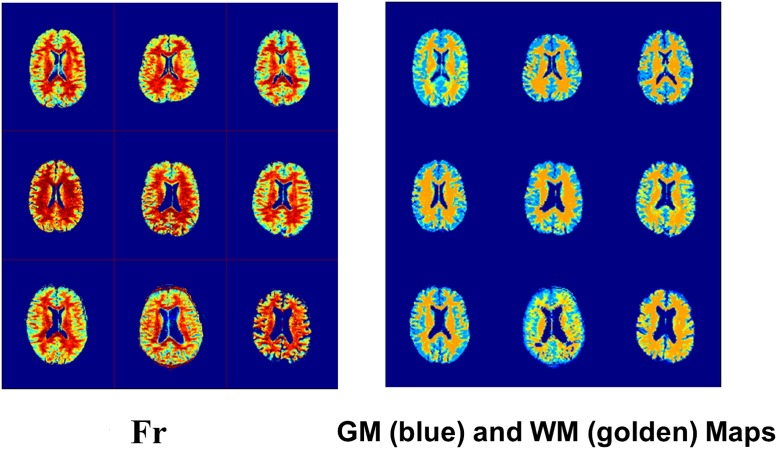
F_r_ maps from Fig. 2b with the GM and WM masks that were obtained from SPM segmentation of the nine subjects.

Fig. 2 displays ZAPPED maps from all nine volunteers of the free proton fraction F_f_ (Fig. 2a) and the restricted proton fraction F_r_ (Fig. 2b), as well as T_2_ maps of the free (Fig. 2c) and restricted exchangeable components (Fig. 2d). The age and sex of the subjects are labeled on the maps. Fig. 3 displays the F_r_ maps from Fig. 2b with the GM and WM masks obtained from segmentation in the nine subjects using SPM. The corresponding numeric results, obtained using the segmentation approach 1 mentioned previously, and calculated average values are presented in Table 1. The mean ultra-short T_2,r_ value in the restricted component is 17.7 ±4.3 μs in GM and 23.4±3.7 μs in WM. The corresponding long T_2,f_ values are 785±58 μs in GM and 671±41 μs in WM. The average Z-spectrum component fractions (mean ± standard deviation) in GM are F_f_ = 0.83±0.02 and F_r_ = 0.17±0.02; in WM, these values are F_f_ = 0.72±0.03 and F_r_ = 0.28±0.03. The percentages of F_f_ and F_r_ in GM are similar for all ages, whereas F_r_ shows a tendency to decrease with age in WM (linear regression: F_r_ = 0.355–0.00165*age, *R* ^*2*^ = 0.656, *p* = 0.008) among healthy subjects.

**Table 1:** Calculated fractions F_f_, F_r_ and T_2_ values T_2,f_, and T_2,r_ in GM (top) and WM (bottom) in nine healthy subjects.

Subject GM	Gender	Age	F_f_	T_2,f_ (μs)	F_r_	T_2,r_ (μs)
1	M	33	0.83	804	0.17	11.7
2	M	35	0.82	815	0.18	18.4
3	F	36	0.84	698	0.16	23.5
4	F	38	0.79	789	0.21	13.5
5	M	48	0.83	763	0.17	14.1
6	M	51	0.86	804	0.14	19.1
7	M	55	0.83	699	0.17	21.5
8	M	59	0.85	809	0.15	15
9	M	71	0.82	880	0.18	22.3
**Mean**		**47.3**	**0.83**	**785**	**0.17**	**17.7**
**SD**		**13**	**0.02**	**58**	**0.02**	**4.3**

Subject WM	Gender	Age	F_f_	T_2,f_ (μs)	F_r_	T_2,r_ (μs)
1	M	33	0.71	651	0.29	17.6
2	M	35	0.71	688	0.29	22
3	F	36	0.72	623	0.28	28.3
4	F	38	0.69	722	0.31	19.5
5	M	48	0.7	647	0.3	24.4
6	M	51	0.73	652	0.27	26.7
7	M	55	0.72	623	0.28	27.2
8	M	59	0.76	719	0.24	20.8
9	M	71	0.77	718	0.23	24.5
**Mean**		**47.3**	**0.72**	**671**	**0.28**	**23.4**
**SD**		**13**	**0.03**	**41**	**0.03**	**3.7**

**Fig 4:**
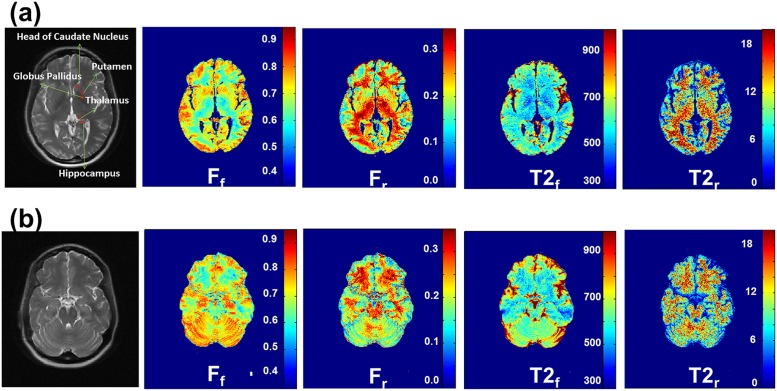
ZAPPED maps of F_f_, F_r_, T_2,f_, and T_2,r_ in the two slices through the basal ganglia and cerebellum. Note that excellent contrast and image quality were obtained in the cerebellum region, and a complicated stripe pattern is displayed.

Excellent contrast and image quality were obtained in the two slices through the basal ganglia and cerebellum as well. The ZAPPED maps of the cerebellum region display a complicated pattern of stripes, as shown in Fig. 4. Z-spectrum fractions F_f_, F_r_ and apparent relaxation times T_2,f_, and T_2,r_ were calculated for five locations in deep GM and WM at the basal ganglia level and appear in Table 2. These include the head of the caudate nucleus, globus pallidus, thalamus and hippocampus.

**Table 2:** Z-spectrum fractions F_f_, F_r_ and apparent relaxation times T_2,f_, and T_2,r_ from deep GM and WM in a 38-year-old female volunteer.

	F_f_	T_2,f_ (μs)	F_r_	T_2,r_ (μs)
**Caudate**	0.80	599	0.20	14.5
**Pallidus**	0.72	537	0.28	20.9
**Thalamus**	0.74	550	0.26	19.2
**Hippocampus**	0.79	599	0.21	16.3
**White Matter**	0.70	641	0.30	22.1

**Fig 5:**
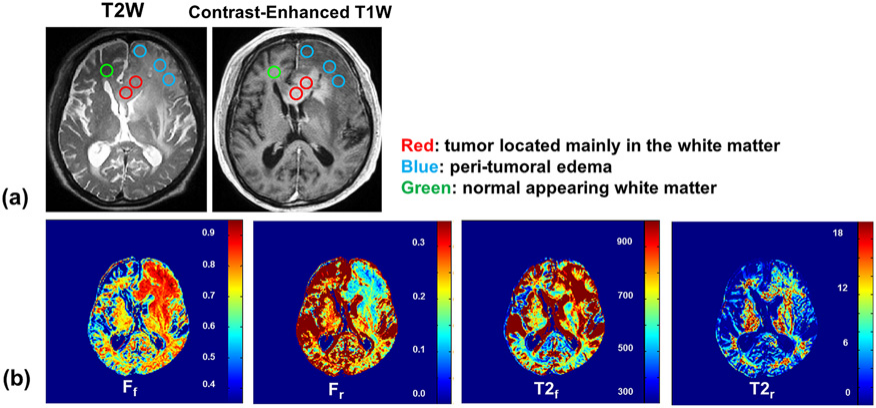
(a) Malignant lymphoma images with ROIs. (b) ZAPPED maps of F_f_, F_r_, T_2,f_, and T_2,r_.

**Fig 6:**
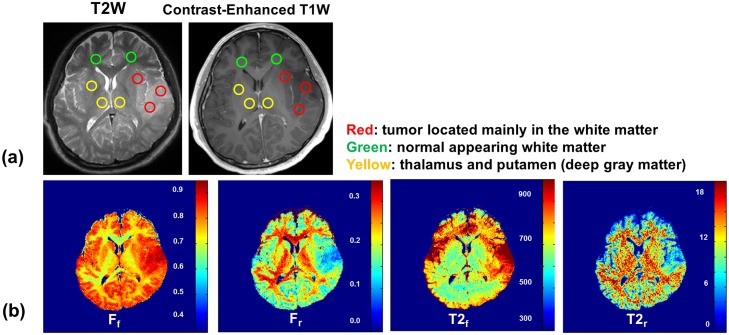
(a) Low-grade glioma images with ROIs. (b) ZAPPED maps of F_f_, F_r_, T_2,f_, and T_2,r_.

The ZAPPED maps of two patients, one with a malignant lymphoma and the other with a low-grade glioma, are presented in Figs. 5 and 6, respectively. Table 3 summarizes the results: long T_2,f_ and ultra-short T_2,r_ values as well as the relative free and restricted fractions in tumor and in normal GM and WM tissues. In the malignant lymphoma case, the lymphoma tissue in WM has a longer T_2,r_ value of 30.6 μs compared to 20.5 μs in normal WM tissue. The fraction of the restricted exchangeable component (F_r_) in WM lymphoma tissue is also smaller (0.16) than that in normal WM tissue (0.26). An area of edema in the surrounding gray matter has a similar T_2,r_ value as that in normal GM tissue. However, F_r_ is smaller in the edema area (0.10) than in normal GM tissue of healthy volunteers (0.17), as shown in Tables 1 and 3.

**Table 3:** Z-spectrum fractions F_f_, F_r_ and T_2_ components T_2,f_, and T_2,r_ in ROIs from brain tumor, edematous GM, normal GM, and normal WM in malignant lymphoma patient (left) and low-grade glioma patient (right).

ROIs of malignant lymphoma	F_f_	T_2,f_ (μs)	F_r_	T_2,r_ (μs)	ROIs of low-grade glioma	F_f_	T_2,f_ (μs)	F_r_	T_2,r_ (μs)
**WM-tumor**	0.84	873	0.16	30.6	**WM-tumor**	0.91	948	0.09	18.1
**WM-normal**	0.74	812	0.26	20.5	**WM-normal**	0.74	826	0.26	28.1
WM-mean*	0.81	916	0.19	19.2	WM-mean*	0.77	743	0.23	23.9
**GM-edema**	0.9	928	0.1	19	**GM-deep**	0.79	665	0.21	19.9
GM-mean*	0.88	949	0.12	26.1	GM-mean*	0.86	817	0.14	22.4

ROI locations appear in Figs. 3 and 4.

* The WM/GM-mean values are the sum of ZAPPED values in the WM/GM component of the whole slice divided by the total number of WM/GM voxels.

In the low-grade glioma case (Fig. 6), the tumor tissue in WM (red ROIs) has a smaller T_2,r_ value (18.1 μs) than that of normal WM (green ROIs) (28.1 μs); in addition, F_r_ in the tumor is much smaller (0.09) than in normal white matter tissue (0.26). Deep GM structures such as the thalamus and putamen have F_r_ values similar to those found in the normal GM of healthy volunteers.

## Discussion

Unlike the existing literature, in which magnetization transfer and z-spectra of two pools are analyzed using kinetic models and the Bloch equation, we adopted a simplified and straightforward approach in this work and used a two-Lorentzian Z-spectrum fitting model, named Z-spectrum Analysis Provides Proton Environment Data (ZAPPED), to map free and restricted exchangeable protons and create images with tissue-sensitive contrast. We have successfully demonstrated that this acquisition method and its analysis scheme are capable of depicting fractions of long T_2,f_ and ultra-short T_2,r_ exchangeable proton compartments, plus their correspondingT_2,f_ and T_2,r_ maps in human GM and WM. Note that the non-exchangeable water proton compartment that is unaffected by MT was neglected in our analysis because CSF was segmented out.

The apparent restricted-pool relaxation times (T_2,r_) estimated for the nine healthy subjects are similar to values reported in the literature [10], but ZAPPED T_2,f_ values are shorter, in part because the effective Z-spectrum frequency resolution as defined by the saturation pulse bandwidth puts a lower limit on fitted Lorentzian linewidths. Furthermore, as stated in the Methods section, T_2,f_ in the ZAPPED model is influenced not only by the traditional T_2_ of non-exchangeable protons, but also by multiple other factors, such as T_1_ and the proton exchange rate between the two pools.

Sled & Pike obtained T_2,f_ values from their Z-spectrum model fits, but they also measured T_2_ directly using a multi-echo sequence [10]. They found that the two sets of numbers are comparable: e.g., 55–56 ms vs. 92–93 ms respectively in GM. However, the multi-echo "observed" values are always a bit longer. Our T_2,f_ values are much shorter (0.785 ms for GM), but this is largely the result of our measurement scheme, which traces out the Z-spectrum with saturation pulses that have a ~400 Hz bandwidth, as stated previously. It's a fairly blunt instrument for examining the true width of the central peak. Our T_2,f_ values thus offer limited clinical insight; however, we focus on the restricted pool quantities T_2,r_ and F_r_. Moreover, we look at relative values instead of absolute values: these provide the contrast in ZAPPED images and help to differentiate the relative contributions from different tissue components.

It is also noteworthy to point out that there are minor differences between the right and left sides of the brain in our T_2_ maps. These could be caused by many different factors, such as CSF partial volume effects, human anatomy, or asymmetry in the display due to a tilted slice orientation. The differences may also be the result of spatially inhomogeneous MT irradiation causing an uneven MT effect in the left and right brain.

The contrast of these maps correlates well with segmented GM and WM regions. The amplitude fraction F_r_ of the restricted environment is non-negligible in both GM (0.17) and WM (0.28). Differences in the restricted movement of exchangeable protons in various neuronal structures may contribute to this observed F_r_ dichotomy between GM and WM. Interestingly, the mean restricted-component T_2,r_ value is shorter in GM than in WM for the nine volunteers, indicative perhaps of a more rigid macromolecular matrix on average in GM; in contrast, the mean value of the free-component T_2,f_ in GM is about 100 μs longer than in WM. In other words, the apparent relaxation properties of the free and restricted components are more separated and distinct in GM than in WM.

The average ZAPPED F_r_ values for WM (0.28) and GM (0.17) are roughly twice the size of the corresponding restricted component fractions determined with other two-pool-based techniques [10, 14,15]. That the numbers are different is not entirely surprising, since ZAPPED estimates the free pool component in a completely different way. However, the *ratio* R of average WM F_r_ values to average GM F_r_ values is comparable to other results: R = 1.65 for ZAPPED vs. R = 1.87 in Ref. 15 and R ~ 1.5 in Ref. 14 (ratio depends on location).

Although the fraction F_r_ of the restricted compartment in GM is similar for all our volunteers, it decreases with age in WM. This trend may likely be attributed to myelin structure changes or decreases in the amount of myelin present. A number of reports have correlated changes in WM with age [16–20]. In particular, Pirpamer et al. [20] have also reported a decrease in the restricted pool fraction in WM as a function of age; moreover, they note that the restricted pool fraction is more sensitive and more specific for age-related effects than the MT ratio. Based on the linear regression parameters presented in Ref. 20, their ratio of F_r_ at age 70 to F_r_ at age 35 is 0.88. The corresponding ratio from our ZAPPED linear regression is 0.81. We believe that this decrease in the restricted component fraction observed in our studies may play an important role in aging and may also be related to the structural changes in WM diseases. Our linear regression was calculated using a limited sample size of nine subjects; a more thorough age-based ZAPPED study with additional subjects will be underway in the near future.

Obtaining Z-spectra from midbrain and cerebellar areas was thought to be difficult due to strong susceptibility effects from air, bone, and soft tissue interactions. However, no obvious artifacts are visible in the ZAPPED images. We found that the distributions of free and restricted components in midbrain and cerebellar structures agree reasonably well with the underlying anatomy. In particular, the stripes visible in the F_f_ map of the cerebellum match the segmentation into GM and WM.

The contrast between WM and GM in MRI images has been investigated by a number of researchers [21–23]. It is known that the relative brightness of adult WM in T_1_-weighted images arises from myelin. Koenig et al. conjectured that cholesterol of myelin was the cause [22]. However, the mechanisms responsible remain unknown.

Neurons consist of cell bodies and axons, mainly located in the GM and WM respectively. In WM, the axons are covered by myelin sheaths. Within the axon, water protons have relatively free movement; in contrast, within the lipid-rich myelin sheath, the water protons may be restricted in movement due to multiple layers of tight sheathing. In GM, neuron cells dominate with much less lipid-like structure than in WM. Water protons thus experience different local environments than those in WM. We believe that the structural and physiological differences in both the free and restricted components between GM and WM contribute to their ZAPPED contrast. The fractions of each component can be quantitatively and easily estimated using the ZAPPED technique.

Our two clinical cases reveal that malignant lymphoma tissue in WM has a longer restricted T_2,r_ value than normal WM tissue, suggesting that the macromolecular pool in abnormal tissue has less solid-like structure. In addition, the restricted compartment fraction F_r_ is smaller in WM lymphoma tissue than in normal WM tissue, similar to the decrease that Yarnkyh [24] observed in a glioma vs. normal WM. The lymphoma results indicate that the T_2,r_ properties of restricted exchangeable protons are changed in tumor, and the proportion of restricted protons is shifted to free exchangeable protons compared to normal WM. In contrast, the T_2,r_ value of edema in areas of GM adjacent to the tumor is comparable to that in normal GM tissue, but the restricted-component fraction F_r_ in the edema is smaller than the value observed in the normal GM of healthy volunteers. Finally, the low-grade WM glioma has a normal T_2,r_ value—comparable to normal WM. These results suggest that ZAPPED may provide useful diagnostic information for tumor imaging, in particular, examining the relationship between tumor grade and ultra-short T_2,r_ values.

Potential clinical applications of ZAPPED, i.e., values and maps for T_2,f_, T_2,r_, F_f_, and F_r_, include assessment of brain changes with age, investigation of myelin diseases, and correlation with tumor staging. However, our preliminary study has several limitations, including a small number of clinical cases and only an approximate estimation of the long free T_2,f_ values due to the convolution of the intrinsic free pool linewidths with the ~400 Hz bandwidth of the saturation pulses used to trace out the Z-spectra. Further clinical studies are required, and optimization of the MT pulse is necessary to improve the sharpness of the ZAPPED results—especially for the narrow free component peak. Though the exact molecular structures of the free and restricted components remain unknown, we believe that basic structural differences are the major contributor to the patterns seen in ZAPPED T_2_ maps and fraction maps in GM and WM, as well as in tumor.

Currently, our model does not incorporate the kinetics of the exchange explicitly. The estimated apparent T_2,f_ values are different from T_2_ values reported in the literature due to the nature of the Z-spectrum fitting technique, as well as influences of non-exchangeable water protons, relative values for T_1_ and T_2_, and the exchange rate. Furthermore, the fraction maps could also depend on the RF power of saturation pulses implemented in the sequence. Despite these limitations, we have demonstrated in this work that ZAPPED produces reasonable maps with good contrast (likely related to myelin content) in healthy subjects and demonstrates good sensitivity to macromolecular loss in glioma and lymphoma. The beauty of this model is that it can generate reasonable contrast and differentiate the fractions and structural properties (such as apparent T_2_) of different components. The ZAPPED model has room for improvement, and further studies will be carried out to enhance and potentially speed up the technique. Options include reducing the number of off-resonance frequencies and/or expanding to multiple slices or a 3D acquisition to cover a large volume of brain using other read-out sequences such as EPI, multi-shot EPI, GRASE, etc. Indeed, preliminary simulation results indicate that reducing the number of frequency sampling points from 59 to 31 would yield similar values and maps for T_2,f_, T_2,r_, F_f_, and F_r_, thereby enabling a scan time reduction from 7:30 to 3:56. This reduced sampling sequence will be put to use soon.

## Conclusion

In summary, we have successfully generated ZAPPED maps of the free and restricted exchangeable components in human GM and WM by fitting the acquired Z-spectra to a phenomenological two-component Lorentzian model with only three independent variables. The fraction maps and apparent T_2_ maps of each component in the ZAPPED analysis can be used to help explain the composition and/or structural differences between GM and WM. The change of the restricted component in WM as a function of age may reflect changes in the restricted proton content within the myelin sheath. Tumor compositions from ZAPPED mapping may indicate differences in the properties of free and restricted components. Further study is required to confirm the age dependence and clinical benefits.
